# Elabela: A Novel Biomarker for Right Ventricular Pressure Overload in Children With Pulmonary Stenosis or Pulmonary Atresia With Intact Ventricular Septum

**DOI:** 10.3389/fcvm.2020.581848

**Published:** 2020-11-12

**Authors:** Jian Wang, Yue Zhou, Qingjie Wang, Bowen Du, Yurong Wu, Qian Chen, Xi Zhang, Yanan Lu, Sun Chen, Kun Sun

**Affiliations:** ^1^Department of Pediatric Cardiology, Xinhua Hospital, School of Medicine, Shanghai Jiao Tong University, Shanghai, China; ^2^Ministry of Education-Shanghai Key Laboratory of Children's Environmental Health, Xinhua Hospital, School of Medicine, Shanghai Jiao Tong University, Shanghai, China; ^3^Clinical Research Unit, Xinhua Hospital, School of Medicine, Shanghai Jiao Tong University, Shanghai, China

**Keywords:** Elabela, pulmonary valvular stenosis, pulmonary atresia with intact ventricular septum, right ventricular afterload, percutaneous balloon pulmonary valvuloplasty

## Abstract

**Background:** Assessing right ventricular overload in children is challenging. We conducted this study involving children with pulmonary valvular stenosis (PS) or pulmonary atresia with intact ventricular septum (PA/IVS) to evaluate the potential of a new endogenous ligand of apelin receptor, Elabela (ELA), as a potential biomarker for right heart overload.

**Methods:** In this prospective cohort study, a total of 118 congenital heart diseases patients with right ventricle outflow tract obstruction were recruited from 2018 to 2019. Among them, 44 isolated PS and 7 PA/IVS patients were selected. Their venous blood was collected, and all patients underwent an echocardiographic examination. Among them, post-operative blood was collected from 24 patients with PS after percutaneous balloon pulmonary valvuloplasty. The plasma ELA concentration was measured using enzyme-linked immunosorbent assay.

**Results:** The ELA was significantly associated with the peak transvalvular pulmonary gradient (*r* = −0.62; *p* = 0.02), thus reflecting the severity of PS or PA/IVS. The ELA significantly increased at 3 days after intervention, when mechanical obstruction of the right outflow tract was relieved. Based on the receiver-operator characteristic curve results, ELA could be a risk factor for duct dependence in patients with critical PS or PA/IVS who are younger than 6 months (AUC: 0.82).

**Conclusion:** ELA concentration and severity of PS or PA/IVS had a significant negative correlation, indicating that ELA might be a novel biomarker for right ventricular afterload and reflect the immediate pressure changes in the right heart. Furthermore, ELA could predict duct-dependency in PS and PA/IVS patients, as valuable as classical echocardiographic indexes.

## Background

Right ventricular afterload has many different causes, including right ventricular outflow tract obstruction (RVOTO), increased pulmonary vascular resistance, abnormal pulmonary artery vascular function, and left ventricular diastolic dysfunction (1). Pulmonary valvular stenosis (PS) and pulmonary atresia with intact ventricular septum (PA/IVS) are congenital heart diseases (CHDs) that comprise approximately 8–12% and 0.7% of all CHDs, respectively (2) They both include obstruction of the right ventricular outflow tract and could result in increased right ventricular pressure, right ventricular hypertrophy, tricuspid valve dysplasia or hypoplasia, and right ventricular hypoplasia, which could also lead to pressure overloaded right ventricular dysfunction, without interference from pulmonary vascular resistance or abnormal vascular function (3, 4).

Assessing right ventricular overload in children is challenging because of the limited non-invasive methods available in clinical practice. An echocardiographic assessment is currently the predominant pre-operative diagnostic test for patients with PS or PA/IVS to assess the location and severity of stenosis and evaluate the morphology and function of the ventricle (5, 6). Furthermore, the lack of clinical symptoms is common in children with congenital pulmonary valve disease during the early stage due to the adaption of the right ventricle to the obstruction of the right ventricular outflow tract. However, long-term untreated obstruction might lead to right ventricular dysfunction and tricuspid regurgitation (7). Therefore, current non-invasive screening tests for right ventricular overload in children have limited diagnostic and prognostic value (8).

Elabela/Toddler (ELA) found by Chng et al., the second endogenous ligand of the apelin receptor (APJ), is a new peptide with 54 amino acids (9–12). The gene encoding ELA with the symbol “*APELA*” assigned by the HGNC, located on chromosome 1, contains three exons (10, 13). ELA contains a secretory signal peptide and a mature region of 32 amino acids (14). So far, ELA was found to be localized in the endothelium of adults, human stem cells, and the kidneys (12). Several studies also showed that ELA was associated with vasodilation, myocardial contraction, and PAH in animal models (15–17). Yang et al. found that the expression of ELA was downregulated in human lung tissues with PAH, and that ELA administration had beneficial effects on cardiac function and remodeling in rat models of PAH (17). Apelin, another endogenous ligand of APJ was also reported that patients with pulmonary arterial hypertension (PAH) have lower apelin levels in plasma (18, 19). However, the function of plasma ELA in humans with right ventricle overload remained unclear. PS and PA/IVS are one form of right ventricular overload, with no interference from pulmonary vascular resistance or abnormal vascular function. Therefore, in this study we chose young patients with either PS or PA/IVS whose right ventricular systolic pressure increased to overcome the increasing impedance to explore the associations among morphological severity, right ventricular overload, and ELA concentration in plasma (20).

## Methods

### Study Design and Participants

**Figure 1:**
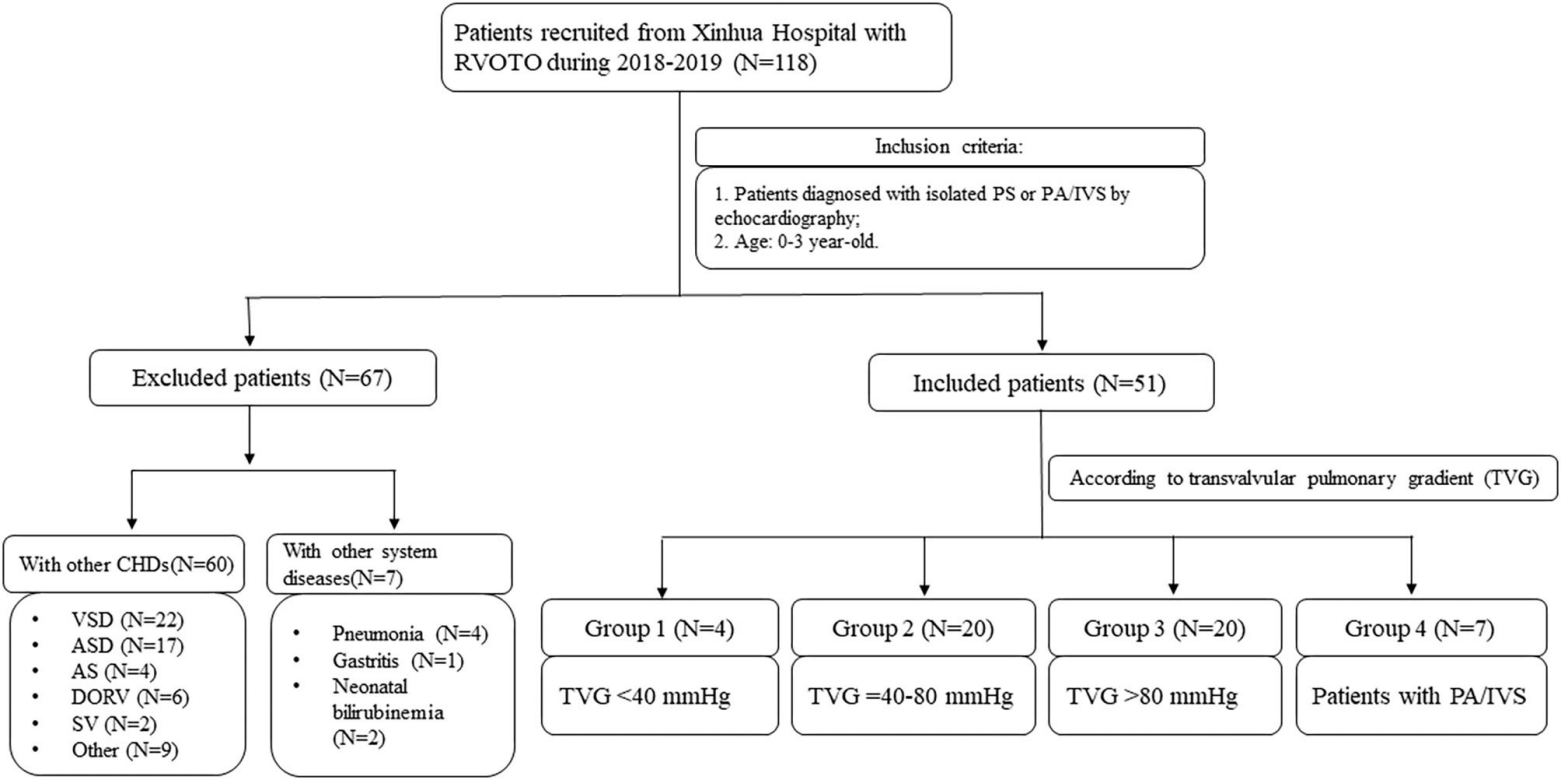
The flow chart of this prospective cohort study. AS, aortic stenosis; ASD, atrial septal defect; CHD, congenital heart disease; DORV, double outlet right ventricle; PS, pulmonary valvular stenosis; PA/IVS, pulmonary atresia with intact ventricular septum; RVOTO, right ventricle outflow tract obstruction; SV, single ventricle; VSD, ventricular septal defect.

The flow chart of this study was shown in Figure 1. From 2018 to 2019, 118 patients diagnosed with CHDs with RVOTO were admitted to Xinhua Hospital affiliated with Shanghai Jiaotong University. Patients were then selected according to the inclusion criteria as follows: (1) isolated PS or PA/IVS diagnosed by echocardiography; (2) 0–3-year-old and exclusion criteria which included patients with neither other CHDs nor diseases of other systems. Finally, a total of 44 isolated PS patients and 7 PA/IVS patients were included in our study. We also collected blood samples of 16 healthy children as control group with the same age of patients in this study. The study was approved by the ethical committee of Xinhua Hospital (XHEC-C-2019-083). Informed consent was obtained from all participants' parents.

At the time of hospital admission, the specific medical history was recorded, including the general condition and course of disease. Information about basic patient characteristics, including age, sex, weight, blood pressure (BP), and birth history were also collected. Previous studies indicated that the severity of RVOTO was usually classified based on the transvalvular pulmonary gradient (TVG); however, no accurate standards apply to children (21–23). Therefore, we divided all patients who underwent an echocardiography examination into four groups according to RVOTO severity: patients with mild PS (TVG <40 mmHg, *n* = 4), moderate PS (TVG = 40–80 mmHg, *n* = 20), severe PS (TVG >80 mmHg, *n* = 20), and PA/IVS (*n* = 7).

### Echocardiography

Echocardiography examinations of all patients were performed by the same sonographer using ultrasound system E8 with 3.5- or 5-MHz sector probes (GE Medical Systems, Bucks, UK) according to the guidelines (24). Pulmonary valve (PV) diameters were measured at end-systole. The mitral valve (MV) diameters, tricuspid valve (TV) diameters, left ventricle widths and lengths, and right ventricle widths and lengths were measured in the standard apical four-chamber view at end-diastole. Additionally, we computed *z*-scores for valve dimensions based on body surface area (BSA) (http://hdb.nbscn.org/zscore). TVG across the PV was calculated using the modified Bernoulli equation along with the spectral Doppler velocities proximal and distal to the valve (25). Right ventricular systolic pressure (RVSP) was usually estimated by the peak pressure gradient between the right ventricle and pulmonary artery using continuous-wave Doppler (26). In this study, RVSP assessments were performed indirectly using the TVG (27, 28). Color and pulsed-wave Doppler were recorded across the cardiac valves and interatrial septum and in the ductus arteriosus (DA).

### Blood Sampling and ELA Measurements

Preoperative venous blood was collected from all patients at the admission and control group. Among them, venous blood of 24 patients was collected at 3 days after percutaneous balloon pulmonary valvuloplasty (PBPV). Whole blood was collected by venipuncture, and stored and preserved in an anticoagulant tube with ethylene diamine tetraacetic acid (EDTA). Within 2 h, these blood samples were centrifuged at 3,000 rpm/min for 15 min. For further use, the supernatant was collected and stored at −80°C.

To determine the concentration of ELA peptide in plasma, samples were measured in duplicate using a commercial enzyme-linked immunosorbent assay (ELISA) kit (Peninsula Laboratories International, Inc., USA).

### Percutaneous Balloon Pulmonary Valvuloplasty

All patients with moderate or severe PS and PA/IVS underwent PBPV. PBPV procedures were performed under general anesthesia (29, 30). To assess the disease, preoperative echocardiography was performed. Iodixanol injection was used as the contrast medium. During catheterization, the pulmonary annulus and orifice were measured to assess the PV morphology. We selected adaptable inflated balloons (Cordis, USA) based on the pulmonary annulus diameter (29). Echocardiography was performed immediately after the operation of PVPB, and 3 days after the operation.

### Statistical Analysis

Data was analyzed using SPSS 19.0 software program (IBM, Inc., USA). Normally distributed data and skewed data were expressed as the mean with standard deviation (SD) or median with interquartile range (IQR), respectively. Continuous variables were tested for distribution normality and variance homogeneity using the Shapiro–Wilk test and Levene test, respectively. These variables were compared using a one-way analysis of variance (ANOVA). Categorical data were expressed as a percentage and compared using the chi-square test. The Kruskal–Wallis test was used as appropriate to compare between-group differences of skewed data.

Correlation between ELA and variables related to PS or PA/IVS were explored using Pearson's correlation analysis. Regression models adjusted with different variables were performed to further explore the association between ELA with TVG and conventional echocardiographic measurements. Moreover, the association between preoperative and postoperative ELA concentrations and TVG was analyzed by using the paired *t*-test.

A duct-dependent CHD in infants was defined as the presence of a critical CHD but good general condition during the fetal period and deterioration when the DA closed after birth. For infants with duct-dependent CHDs, it was critical to maintain the duct opening by using prostaglandin E (PGE) after birth (31). To determine a cutoff point of ELA for DA maintenance in young infants under 3-month old, the receiver-operator characteristic (ROC) curve analysis was performed. In this study, *p* < 0.05 was considered statistically significant.

## Results

### Basic and Echocardiographic Characteristics

Basic characteristics and echocardiography measurements of different groups are described in Table 1, and the basic characteristics of the control group are described in Supplement 1. Most patients with severe PS or PA/IVS were neonates or young infants. Due to disease severity, weight, systolic blood pressure (SBP), and diastolic blood pressure (DBP) in PA/IVS group were significantly lower than those in other groups, and preoperative pro-BNP was higher when the disease was more severe (*p* = 0.001). More patients with severe PS and PA/IVS was born from a mother with parity >2 (*p* = 0.04); similarly, more patients with severe PS and PA/IVS had a mother with a history of spontaneous abortion (*p* = 0.04), but no history of CHD. In addition, we also searched the information whether these patients underwent the re-intervention or re-operation due to the restenosis after the operation. We found that there was no statistically significant difference between the four groups about re-operation (*p* = 0.088). Because the indications of the re-intervention or re-operation were still controversial, the decision of re-operation depended mostly on the postoperative TVG, development of right ventricle and pulmonary annulus.

**Table 1:** Basic and echocardiographic characteristics of different groups including all subjects.

	**PS**			**PA/IVS**	
	**<40 mmHg *N* = 4**	**40–80 mmHg *N* = 20**	**>80 mmHg *N* = 20**	***N* = 7**	***p*-Value**
**Basic characteristics**					
Age (months)	18.00 ± 6.9	11.45 ± 14.7	7.6 ± 16.2	0.29 ± 0.5	0.175^a^
Sex (M/F)	2/2	14/6	9/11	6/1	0.185^b^
SBP (mmHg)	**93.80** **±** **21.3**	**89.25** **±** **15.1**	**90.42** **±** **13.7**	**69.14** **±** **4.2**	**0.007^a^**
DBP (mmHg)	**57.40** **±** **9.5**	**48.95** **±** **9.4**	**49.68** **±** **10.6**	**38.43** **±** **4.1**	**0.031^a^**
Weight (kg)	**11.25 (9.7, 12.1)**	**8.38 (6.8, 11.8)**	**5.80 (3.6, 8.7)**	**3.05 (2.7, 3.6)**	**<0.001^c^**
Pro-BNP	**168.95 (57.57, 232.5)**	**261.10 (104.20, 401.30)**	**629.80 (253.20, 1949.00)**	**2476.00 (2223.00, 25316.00)**	**0.001^c^**
LOS (d)	15.25 ± 6.7	7.47 ± 4.5	10.93 ± 5.4	12 ± 4.7	0.244^a^
Parity ≥2 (%)	**1 (25.0)**	**8 (40.0)**	**14 (73.7)**	**6 (85.7)**	**0.036^b^**
Abortion ≥1 (%)	**0 (0)**	**3 (15.0)**	**9 (47.4)**	**4 (57.1)**	**0.035^b^**
Re-operation (%)	0 (0)	2 (10.0)	2 (10.0)	3 (42.9)	0.088^b^
**Echo characteristics**					
TVG (mmHg)	**31.5 (18.8, 38.3)**	**65.0 (48.0, 75.3)**	**108.5 (94.8, 137.5)**	**160.0 (160.0, 160.0)**	**<0.001^c^**
TV *z*-score	**1.03** **±** **2.9**	**1.46** **±** **1.1**	**1.19** **±** **2.2**	**−1.05** **±** **1.3**	**0.026^a^**
TV/MV	**1.26 (1.0,1.6)**	**1.03 (0.9,1.2)**	**1.06 (0.9,1.3)**	**0.78 (0.7,0.8)**	**0.001^c^**
PV *z*-score	**–**1.41 (**–**1.7, 0.1)	**–**1.75 (**–**2.4, **–**0.7)	**–**1.92 (**–**2.7, **–**1.6)	**–**1.96 (**–**2.8, **–**1.3)	0.161^c^
PV/AV	0.86 (0.8, 1.1)	0.78 (0.6, 0.9)	0.74 (0.7, 0.8)	0.6 (0.6, 0.7)	0.094^c^
RV/LV width ratio	**1.07** **±** **0.2**	**0.93** **±** **0.2**	**0.93** **±** **0.3**	**0.68** **±** **0.1**	**0.011^a^**
RV/LV length ratio	**0.93** **±** **0.2**	**0.95** **±** **0.16**	**0.84** **±** **0.19**	**0.55** **±** **0.1**	**<0.001^a^**
Duct dependence (%)	**0 (0)**	**3 (15.0)**	**6 (30.0)**	**7 (100.0)**	**<0.001^b^**
**Elabela levels**					
ELA (ng/mL)	**40.69 (39.2, 46.5)**	**17.59 (10.6, 35.7)**	**9.86 (1.8, 24.6)**	**4.85 (1.9, 6.7)**	**0.002^c^**

* *Data are expressed as the mean ± SD or median (interquartile range)*.

*M, male; F, female; SBP, systolic blood pressure; DBP, diastolic blood pressure; BSA, body surface area; LOS, length of stay; TVG, transvalvular pulmonary gradient; TV, tricuspid valve; RV, right ventricle; LV, left ventricle; PV, pulmonary valve; PV/AV, pulmonary valve/aortic valve; DA, ductus arteriosus. Group 1: patients with mild PS (TVG <40 mm Hg). Group 2: patients with moderate PS (TVG = 40–80 mm Hg). Group 3: patients with severe PS (TVG >80 mm Hg). Group 4: patients with PA/IVS. Data were expressed as the mean ± SD for normally distributed data or median with 25th and 75th quartiles for skewed data. Bold values were variables with P-value < 0.05*.

a *One-way ANOVA tests*.

b *Chi-square test*.

c *Kruskal–Wallis test*.

Detailed echocardiographic examination results were demonstrated in Table 1. The median TVG values of the four groups were 31.5, 65.0, 108.5, and 160 mmHg, respectively. Although there was no forward blood flow in patients with PA/IVS, maximum TVG was used for this study for the convenience of statistical calculations. Additionally, other variables related to right ventricular morphological characteristics had significant inter-group differences, including the TV *z*-score (*p* = 0.03), TV/MV (*p* = 0.001), and right ventricle/left ventricle width ratio (*p* = 0.01) and length ratio (*p* < 0.001); these were also in accordance with disease severity.

### Correlation of ELA With TVG

**Figure 2:**
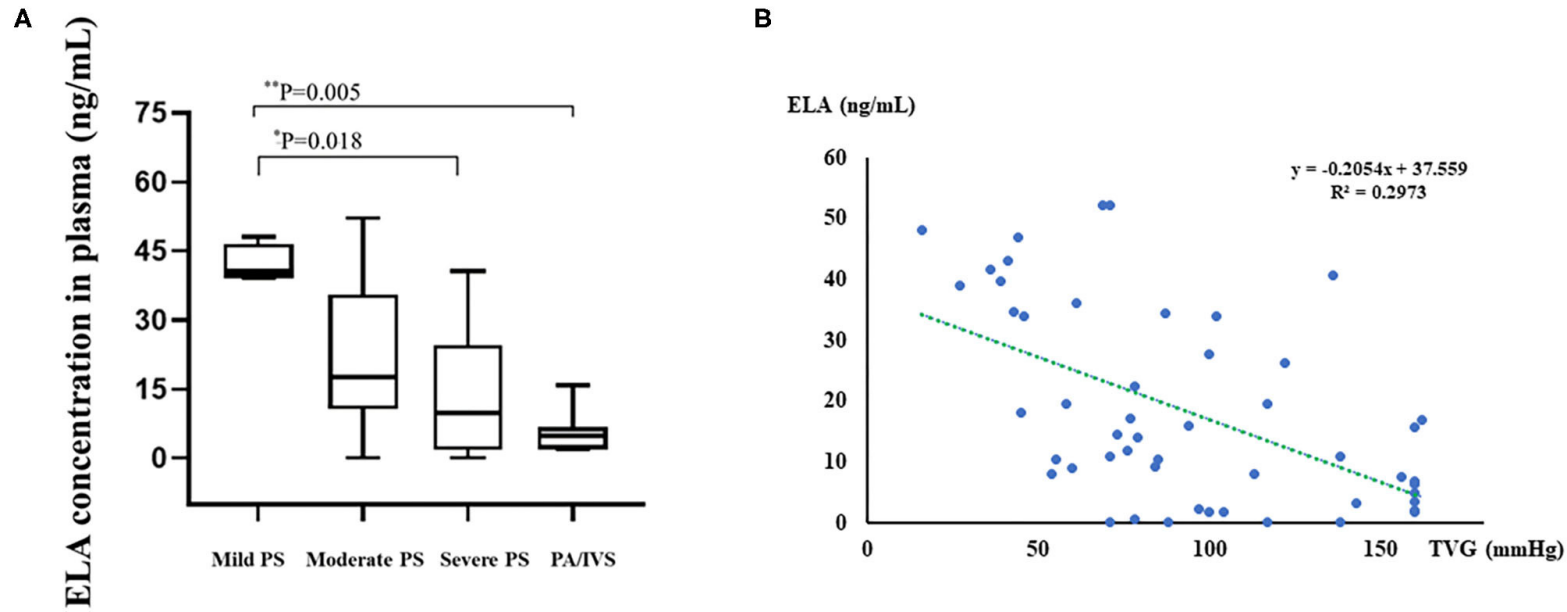
Boxplot of plasma ELA levels of all groups and linear regression analysis between preoperative TVG and ELA concentrations. **(A)** Boxplot of plasma ELA levels (median; IQR) of all groups. **(B)** The linear regression analysis between TVG and ELA concentration. Mild PS: TVG < 40 mmHg; moderate PS: TVG = 40–80 mmHg; severe PS: TVG > 80 mmHg. *0.01 < *p* < 0.05; **0.001 < *p* < 0.01.

Based on the ELISA data, the median ELA concentrations of the four groups were 40.69, 17.59, 9.86, and 4.85 ng/mL, respectively (Table 1, Figure 2A). There was a statistically significant difference among groups (*p* = 0.02). According to the pair-wise comparison, significant differences in ELA levels were observed between patients with mild PS and severe PS (*p* = 0.018) and patients with mild PS and PA/IVS (*p* = 0.005). As shown in Figure 2B, we found there was the negative correlation between pre-operative TVG and ELA concentration (*r*^2^ = 0.2973), that is, ELA concentration was lower when TVG was greater. These results indicated that ELA was a potential biomarker for preoperative TVG. In addition, we also tested the ELA concentration of the control group and compared with PS group. The result showed that ELA concentration of PS patients were higher than control group (Supplement 2, *p* = 0.001). We found that the ELA concentration in the control group was lower than that in the disease group. This result might be due to the unpaired age, small sample size, or other underlying factors related to ELA. However, it has no interference with the conclusion of the association between ELA and TVG. Thus, we put this result in the Supplementary Material.

**Figure 3:**
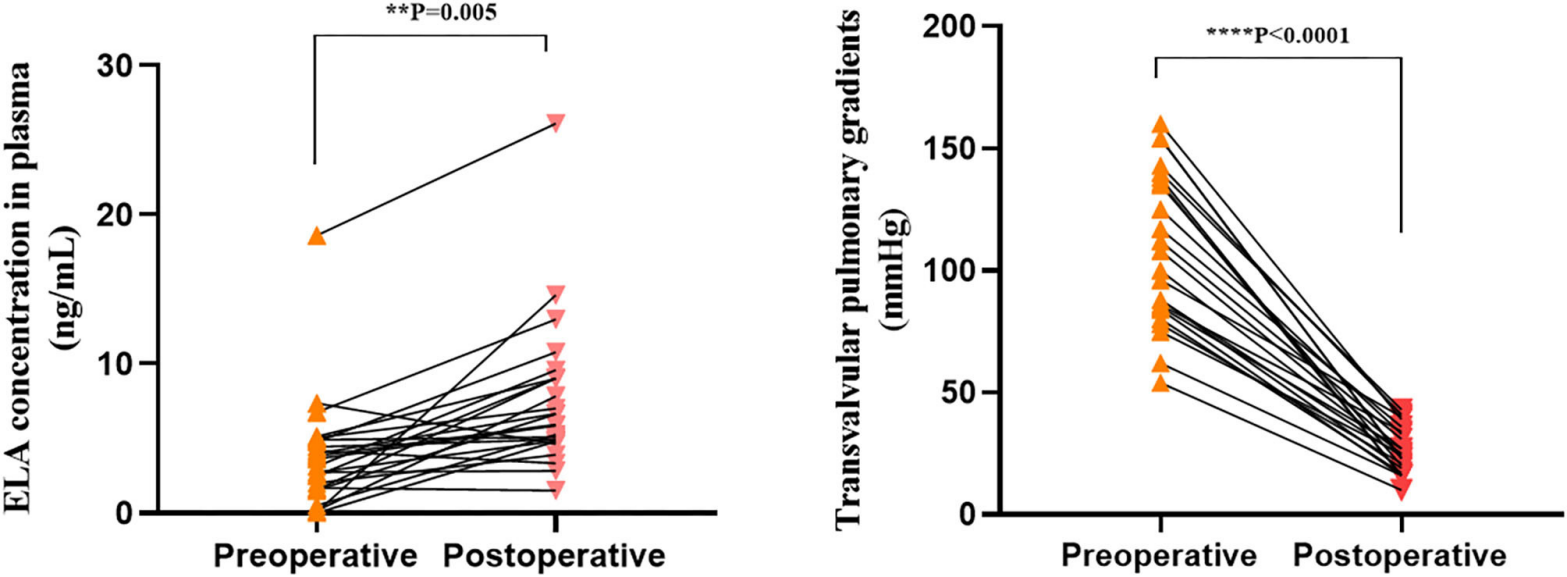
Preoperative and postoperative transvalvular pulmonary gradients (TVG) and ELA concentrations. **0.001 < *p* < 0.01; ****p < 0.0001.

We collected postoperative (3 days after intervention) plasma from 24 patients with severe PS and PA/IVS who underwent PBPV in order to measure ELA concentrations in the same individuals with different right ventricle loading conditions. As shown in Figure 3, we compared preoperative and postoperative change of ELA with the change of TVG of 24 patients. Among them, the ELA concentrations of 19 patients increased greatly with decreasing TVG after alleviation of the right ventricular outflow tract. There was statistical difference between the preoperative and postoperative groups regarding TVG (*p* < 0.001) and ELA concentrations (*p* = 0.005). We had the correlation analysis between postoperative TVG and ELA concentration, and found that there might not exist a correlation between these two variables (*r*^2^ = 0.0297, Supplement 3).

**Figure 4:**
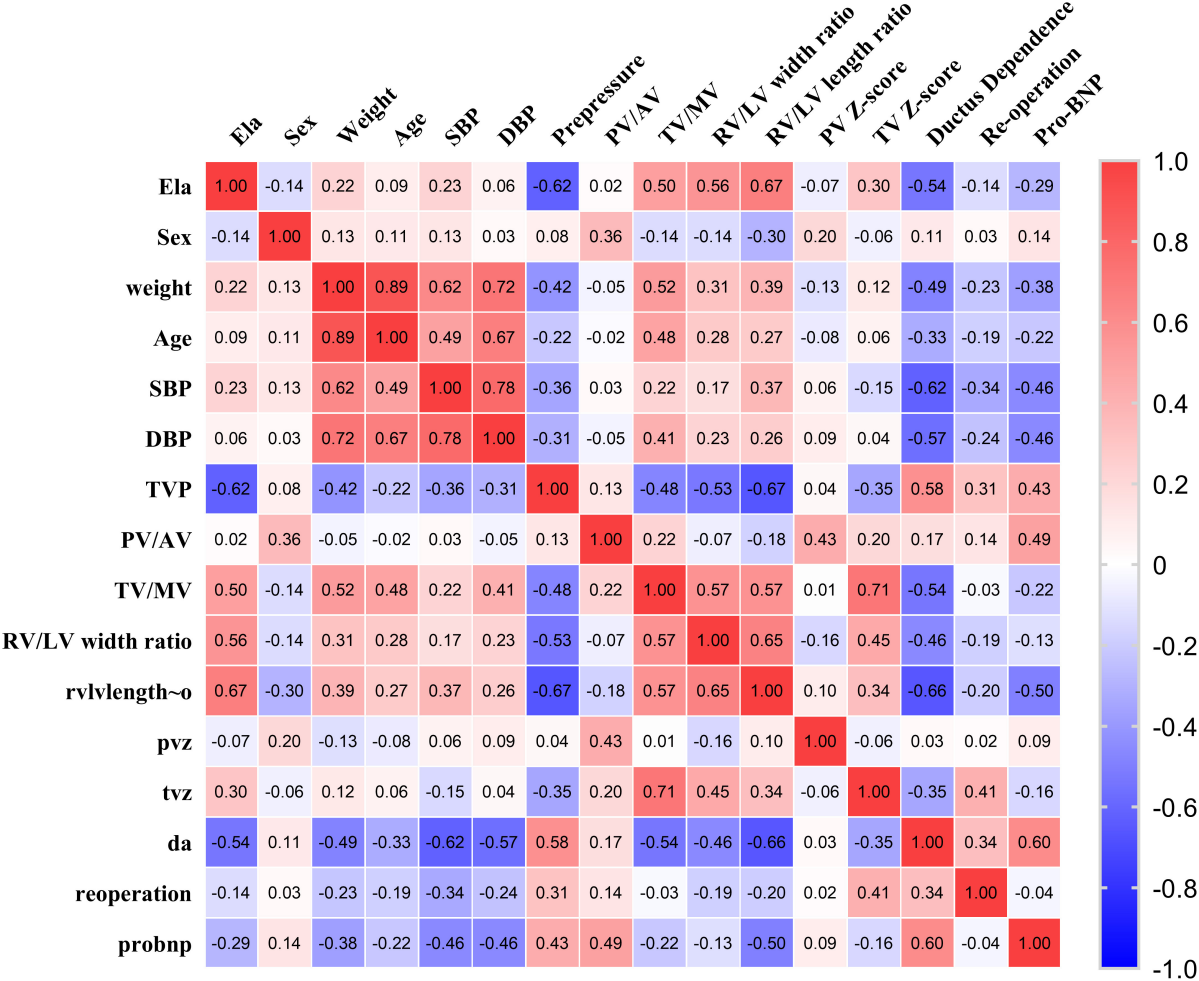
The hot spot of correlation coefficients of ELA and demographic and echocardiographic measurements.

Moreover, the relationships between ELA concentrations and other variables, including demographic information and echocardiographic characteristics, were also analyzed. The correlation coefficients between variables are shown in Figure 4 (the hot spot). We found that ELA was significantly negatively correlated with preoperative TVG (*p* = −0.62; *p* = 0.02) and ductus dependence in young infants (*r* = −0.54; *p* = 0.001). Additionally, ELA was found to be positively correlated with important morphological assessments of the right ventricle, including TV/MV (*r* = 0.50; *p* = 0.03) and right ventricle/left ventricle width ratio (*r* = 0.56; *p* = 0.001) and length ratio (*r* = 0.67; *p* < 0.001).

To further explore the association between ELA and preoperative TVG, regression analysis was performed. During the first regression analysis (Table 2, model 1), ELA was the independent variable and TVG was the dependent variable. In model 1, ELA was significantly negatively relevant to TVG (coefficient: −1.44; *p* < 0.001). In the second regression analysis (Table 2, model 2), after adjustment of age, sex, weight, SBP, DBP, and pro-natriuretic peptide (pro-BNP), ELA was still negatively associated with TVG significantly (coefficient: −1.01; *p* = 0.001). In the third regression analysis (Table 2, model 3), after additional adjustment of right ventricle/left ventricle length ratio, which were classical echocardiographic measurements of right ventricular development, the *p*-value of regression increased by 0.151 with a coefficient of −0.61, which might illustrate the association between ELA and echocardiographic measurements of right ventricle/left ventricle width or length ratio. Likewise, taking echocardiographic measurements as dependent variables, different regression models were then performed. It was found that, ELA was significantly relevant to both right ventricle/left ventricle width ratio (coefficient: 0.008; *p* < 0.001) and right ventricle/left ventricle length ratio (coefficient: 0.007; *p* < 0.001). After adjustment basic characteristics, ELA was still significantly positively associated with right ventricle/left ventricle width ratio (coefficient: 0.007; *p* < 0.001) and right ventricle/left ventricle length ratio (coefficient: 0.003; *p* = 0.001).

**Table 2:** Results of regression models assessing the independent variable of Elabela and its association with transvalvular pulmonary gradient (TVG) and conventional echocardiographic measurements.

	**Coefficient of Elabela**	**95% CI**	***p*-Value**
**TVG (mmHg)**			
Model 1^a^	−1.44	−2.08, −0.81	<0.001
Model 2^b^	−1.01	−1.72, −0.48	0.001
Model 3^c^	−0.61	−1.45, 0.24	0.151
**RV/LV width ratio**			
Model 1^a^	0.008	0.005, 0.011	<0.001
Model 2^b^	0.007	0.004, 0.011	<0.001
**RV/LV length ratio**			
Model 1^a^	0.008	0.004, 0.011	<0.001
Model 2^b^	0.006	0.003, 0.009	0.001

a *Model 1 was only adjusted for the independent variable of ELA*.

b *Model 2 was adjusted for some basic characteristics, including age, sex, weight, systolic blood pressure (SBP), diastolic blood pressure (DBP), and pro-natriuretic peptide (pro-BNP)*.

c *Model 3 was adjusted for additional echocardiographic measurements of RV/LV width ratio and RV/LV length ratio, except for basic characteristics*.

### Receiver-Operation Characteristic Curve

**Figure 5:**
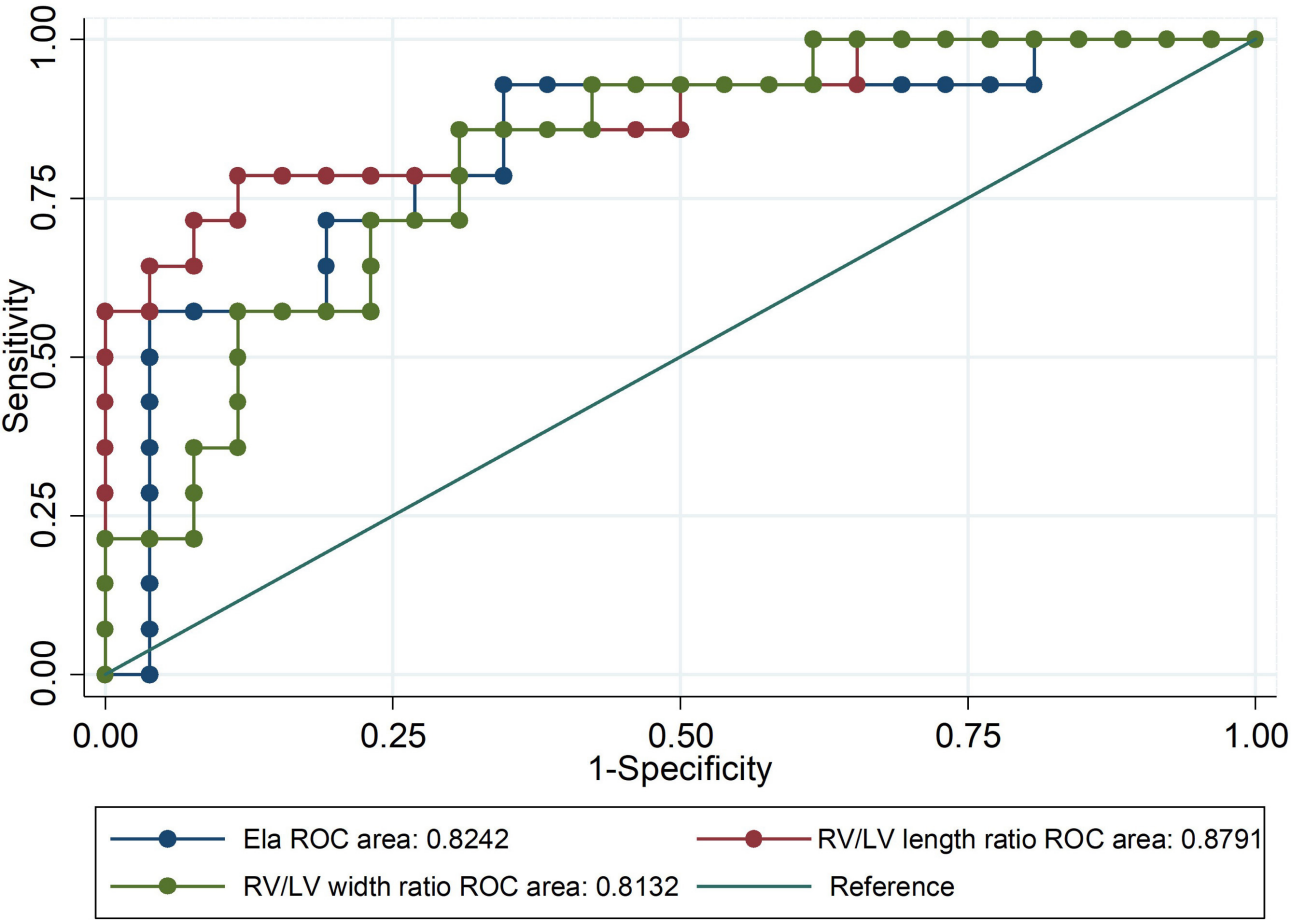
Receiver-operator characteristic (ROC) curve of ELA concentrations and right ventricle (RV)/left ventricle (LV) length ratio and width ratio as predictors of neonatal ductal dependence. Patients with ELA concentrations more than 10.698 ng/mL were more likely to be ductus-independent during the neonatal period.

DA maintenance was important for young infants with critical PS (CPS) and PA/IVS (31). Therefore, ROC curves of the ELA concentration, right ventricle/left ventricle length ratio, and right ventricle/left ventricle width ratio as predictors of neonatal ductal independence were performed. We selected infants younger than 6 months with a history of PGE use during the neonatal period. As shown in Table 3 and Figure 5, patients with ELA concentrations more than 10.698 ng/mL were more likely to have ductus dependence during the neonatal period (specificity, 75.0%). The specificity values of the right ventricle/left ventricle length ratio and right ventricle/left ventricle width ratio were 99.5 and 88.5%, respectively. In addition, AUC of ELA was 0.82, whereas the right ventricle/left ventricle length ratio and right ventricle/left ventricle width ratio were 0.88 and 0.81, respectively.

**Table 3:** Sensitivity and specificity of receiver-operator characteristic (ROC) curve of ELA concentrations and right ventricle (RV)/left ventricle (LV) length ratio and width ratio as predictors of neonatal ductal dependence.

	**Proportion (%)**	**Standard error (±, %)**	**95% CI (%)**
**ELA**			
Sensitivity	63.2	11.1	38.4–83.7
Specificity	75.0	7.7	56.6–88.5
PPV	60.0	11.0	36.1–80.9
NPV	77.4	7.5	59.0–90.4
**RV/LV length ratio**			
Sensitivity	66.7	12.2	38.4–88.2
Specificity	99.5	6.3	69.9–97.6
PPV	76.9	11.7	46.2–95.0
NPV	82.1	7.2	63.1–93.9
**RV/LV width ratio**			
Sensitivity	57.1	13.2	28.9–82.3
Specificity	88.5	6.3	69.8–97.6
PPV	72.7	13.4	39.0–94.0
NPV	79.3	7.5	60.3–92.0

*PPV, positive predictive value; NPV, negative predictive value*.

## Discussion

In this study, the ELA concentration in plasma was significantly negatively correlated with the right ventricular afterload and morphological severity of patients with PS or PA/IVS, thereby indicating that ELA was a potential biomarker for immediate right ventricular overload in children. Furthermore, it could be a predictor for duct-dependent pulmonary circulation during infancy.

ELA was a hormone essential for heart development signals *via* the apelin receptor (13). A previous study showed that ELA prevented pressure overload-induced heart failure, possibly *via* the suppression of angiotensinogen (ACE) expression and pathogenic angiotensin II signaling, in mice (32). However, there has not yet been clinical study about the therapeutic effect of ELA in heart failure. The function of ELA in human heart failure is worth further exploration. It was also reported that the ELA was associated with PAH in a rat model, which was one form of right ventricular overload diseases, and could help improve RV remodeling. The potential mechanism was that ELA, as an endogenous agonist of the human apelin receptor, exhibited cardiovascular functions such as increasing cardiac contractility and cardiac output, thus leading to vasodilatation (17). However, PAH usually had various causes, and the plasma ELA concentration in humans remains unclear. To explore the association between the ELA concentration and right ventricular afterload, we chose patients with PS or PA/IVS, with no interference from the pulmonary vascular resistance.

Our study showed, for the first time, that the ELA plasma concentration was associated with right ventricle overload in patients with PS or PA/IVS. The ELA concentration was significantly negatively related to TVG, indicating that ELA was a new biomarker for assessing the severity of PS and PA/IVS. Furthermore, ELA concentration increased 3 days after PBPV, indicating that ELA was an immediate biomarker for right ventricular afterload, and the correlation between ELA and TVG was inverse. Although the consistency of results was good, it still needed to be proved by further studies due to the small number of samples. So far, ELA was just found localized in the endothelium of adults, human stem cells, and the kidneys (12). Based on our above conclusion, which was that ELA was associated with pressure overload, we hypothesized that ELA might be secreted from the atria or ventricle that are sensitive to changes in pressure, but that would require further study. The ROC curve results indicated that ELA might be a predictor of ductus dependence, like the right ventricle/left ventricle length ratio and the right ventricle/left ventricle width ratio, during the neonatal period. In other words, ELA was probably a valuable predictor of duct dependence in young infants with CPS or PA/IVS. The maintenance of DA was very important for young infants with CPS or PA/IVS. ELA, whose sensitivity and specificity was similar to echocardiographic indexes, was also found to be capable of predicting ductal dependence. Therefore, ELA might be used as an extra serological method to predict DA open in clinical practice. Previous study found that APJ was a static pressure sensitive receptor *via* the PI3K-autophagy pathway (33). Some evidence indicated that apelin/APJ pathway could regulate various important genes (e.g., eNOS, KLF2 and miR-424) to have effect on the pathogenesis of PAH (34). Thus, the reason why ELA was a novel biomarker of assessing right ventricular afterload might be related to its receptor APJ. Although there might be a correlation between ELA level and the underlying defect, we thought this correlation required further study due to the individual variances in ELA concentrations occurred in different populations (35–37). In addition, the correlation of the ELA concentration with long-term right ventricular pressure change and remodeling in human remains unclear; therefore, this is of interest and should be evaluated in future studies. Because of the small sample size, these results still need further validation by well-designed cohort studies.

The assessment of right ventricular function has recently been developed. In clinical practice, right ventricular afterload is most indicated by pulmonary vascular resistance. However, a more comprehensive understanding of afterload should consider not only the pulmonary vasculature but also potential ventricular resistive or outflow components (38). Therefore, patients with PS or PA/IVS were included in our study to explore right ventricular overload exclusive of the interference from pulmonary vascular resistance or abnormal vascular function.

The physiologically sound definition of right ventricular overload should be indicated in both biological and clinical studies (39). At present, the gold standard for hemodynamic assessment is the measurement of RVSP by cardiac catheterization (6). However, this assessment method lacks the suitability of longitudinal monitoring and serial assessment of pressure overload due to its invasiveness (40). In addition, Doppler echocardiography is used as the first-line imaging test to assess right ventricular afterload, which is mainly indicated by the TVG (41, 42).

Trials to detect signs of survival or clinical deterioration usually require a large sample size, which is relatively difficult for low-incidence diseases such as CPS and PA/IVS. Therefore, a right ventricular surrogate endpoint in clinical practice might help determine the ultimate disease prognosis and trial designs because right ventricular dysfunction is one of the leading causes of mortality for these patients. Decreased exercise tolerance is one of the most important prognostic factors for hospitalization or death of patients with right ventricular failure associated with CHD; however, it is difficult to assess in young children or infants (43).

Research to find biomarkers would be helpful for the identification and management of patients with heart failure, but it is mainly focused on the identification of left ventricle dysfunction. Although some biomarkers have been reported to be related to right ventricle dysfunction, their value as prognostic or diagnostic markers remain controversial due to significant individual variance and non-specificity (44). For example, BNP is increased in left cardiac dysfunction (42, 45–47). Recently, it has been reported that BNP levels are also correlated with right ventricular dysfunction in CHD patients (48, 49). However, the value of BNP as a prognostic or diagnostic marker remains controversial and might not be a reliable marker for distinguishing the dysfunction level within each ventricular side (50). Therefore, the ability of current biomarkers to distinguish right heart dysfunction caused by left heart failure and right ventricular intrinsic pathology using biomarkers of load-dependent or load-independent right heart dysfunction needs further improvement (51). Recent study found that the ELA-APJ axis could protect against heart failure induced by pressure overload (32). Although the evidence to think that ELA is a biomarker to assess right heart dysfunction is not enough, our results has provided a new perspective for the evaluation of right ventricular function.

## Strengths and Limitations

In this study, we first found that ELA was associated with right ventricular afterload and has the potential to be a novel biomarker for assessing right ventricular afterload. However, this study had some limitations. First, the sample size was relatively small. Second, the correlation among the ELA concentration and long-term right ventricle remodeling and loading changes remain unclear. Third, in our study, the ELA concentration in the control group was lower than that in the disease group, which might be due to the unpaired age, small sample size, or other underlying factors related to ELA. Since the large individual variances of ELA concentrations, its concentration in healthy children requires further study (35–37).

## Conclusion

In this study, the plasma ELA concentration was significantly associated with the severity of PS or PA/IVS. The significantly negative correlation between ELA and TVG indicated that ELA was probably a novel indicator of the immediate response of right ventricular pressure overload, and the correlation between ELA and TVG was found to be inverse. The clinical value of ELA for assessing right ventricular pressure overload needs further validation in well-designed clinical study, and its mechanism also needs further investigation.

## Data Availability Statement

The raw data supporting the conclusions of this article will be made available by the authors, without undue reservation.

## Ethics Statement

The studies involving human participants were reviewed and approved by the ethical committee of Xinhua Hospital (XHEC-C-2019-083). The individual(s), and/or minor(s)' legal guardian/next of kin provided their written informed consent to participate in this study.

## Author Contributions

KS, SC, and YL contributed equally to the study, conceived and designed the study, collected data. JW and YZ prepared an analytical plan, analyzed data, and drafted the initial manuscript. QW and BD were involved in data collection. YW made the echocardiography examinations. QC and XZ collaborated in the revision and interpretation of the data and results. All authors reviewed and revised the manuscript, and approved the final manuscript as submitted and agreed to be accountable for all aspects of the work.

## Conflict of Interest

The authors declare that the research was conducted in the absence of any commercial or financial relationships that could be construed as a potential conflict of interest.
