# Supplementary material for: Elabela: A Novel Biomarker for Right Ventricular Pressure Overload in Children With Pulmonary Stenosis or Pulmonary Atresia With Intact Ventricular Septum
Source: Front Cardiovasc Med. 2020 Nov 12;7:581848. doi: 10.3389/fcvm.2020.581848 (PMC7688667; doi:10.3389/fcvm.2020.581848)

Supplement 1. Basic characteristics of disease and control groups including all subjects.

|  | PS and PA/IVS  N=51 | Control  N=16 | *P*-value |
| --- | --- | --- | --- |
| **Basic characteristics**  Age (months) | 8.92 ± 2.0 | 32.94 ± 2.8 | <0.001^a^ |
| Sex (M/F) | 31/20 | 6/10 | 0.102^b^ |
| SBP (mmHg) | 87.88 ± 2.2 | 91.80 ± 1.5 | 0.343^a^ |
| DBP (mmHg) | 48.42 ± 1.4 | 54.93 ± 1.9 | 0.023^a^ |
| Weight (kg) | 7.40 (3.7, 10.8) | 14.95 (13.4, 16.3) | <0.001^c^ |
| Pro-BNP | 394.80 (144.50, 1808.00) | 115.80 (98.91, 226.70) | 0.004^c^ |
| Parity >2 (%) | 29 (58.0) | 8 (50.0) | 0.575^b^ |
| Abortion >1 (%) | 16 (32.0) | 3(18.8) | 0.308^b^ |
| **Elabela levels** |  |  |  |
| ELA (ng/mL) | 14.01 (4.8, 34.1) | 4.02 (3.0, 6.3) | 0.001^c^ |

* Data are expressed as the mean ± SD or median (interquartile range). M: male; F: female; SBP: systolic blood pressure; DBP: diastolic blood pressure. Data were expressed as the mean ± SD for normally distributed data or median with 25th and 75th quartiles for skewed data.

^a^ One-way ANOVA tests. ^b^ Chi-square test. ^c^ Kolmogorov-Smirnov test.

Supplement 2. The ELA concentration in plasma of PS and control groups. PS: pulmonary valvular stenosis. ^***^p<0.001.

Supplement 3. The linear regression analysis between postoperative TVG and ELA concentrations.


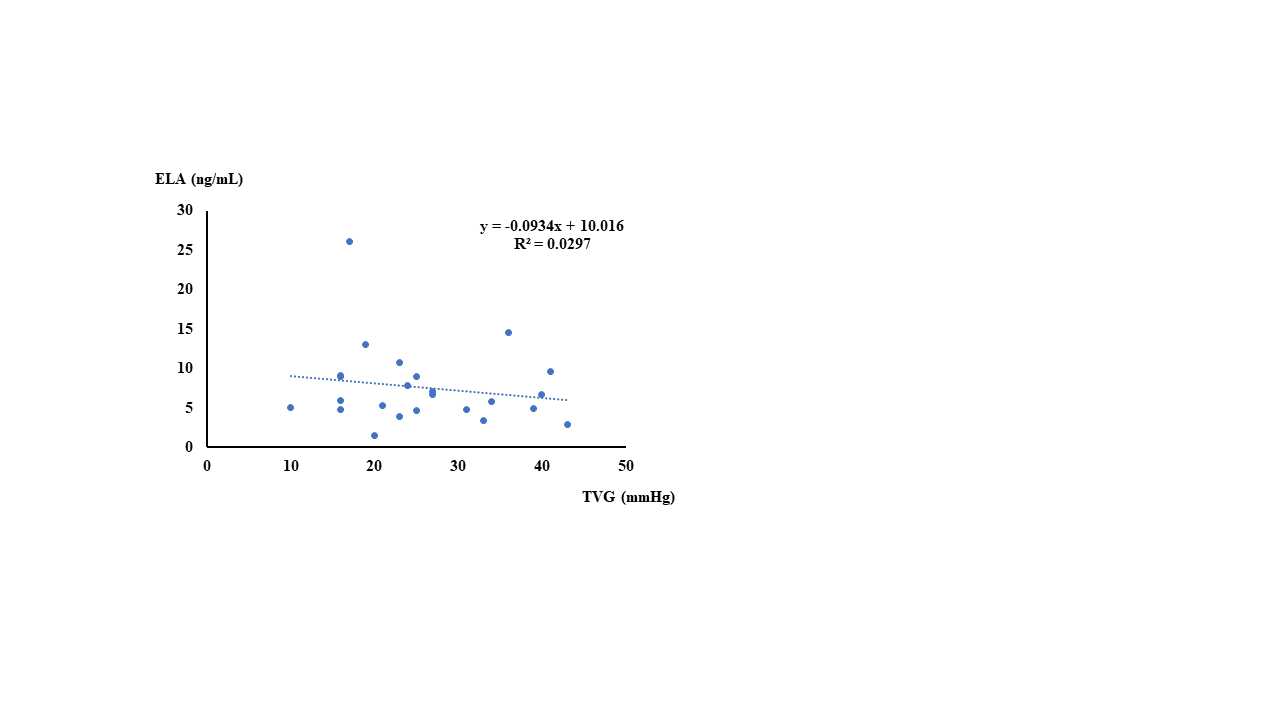

Supplement: Supplementary file 1 [file Table_1.DOCX]
